# Supplementary material for: Metronomic Chemotherapy Modulates Clonal Interactions to Prevent Drug Resistance in Non-Small Cell Lung Cancer
Source: Cancers (Basel). 2021 May 7;13(9):2239. doi: 10.3390/cancers13092239 (PMC8125381; doi:10.3390/cancers13092239)
Supplement: Supplementary file 1 [file cancers-13-02239-s001.zip › cancers-1184890-supplementary.pptx]

## Slide 1
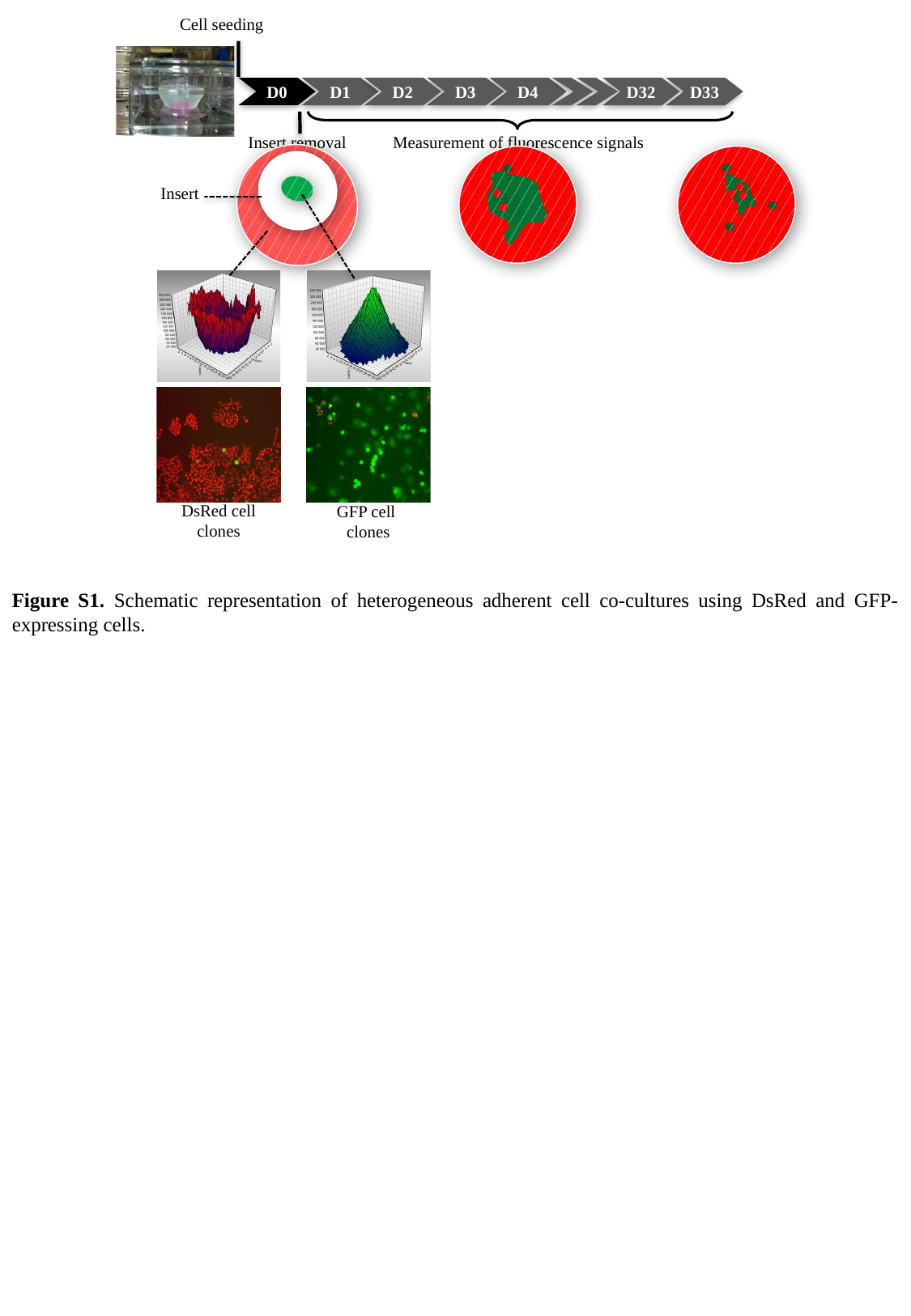

Cell seeding
D0
D3
D4
D32
D33
D1
D2
Insert removal
Measurement of fluorescence signals
Insert
DsRed cell clones
GFP cell
clones
Figure S1. Schematic representation of heterogeneous adherent cell co-cultures using DsRed and GFP-expressing cells.

## Slide 2
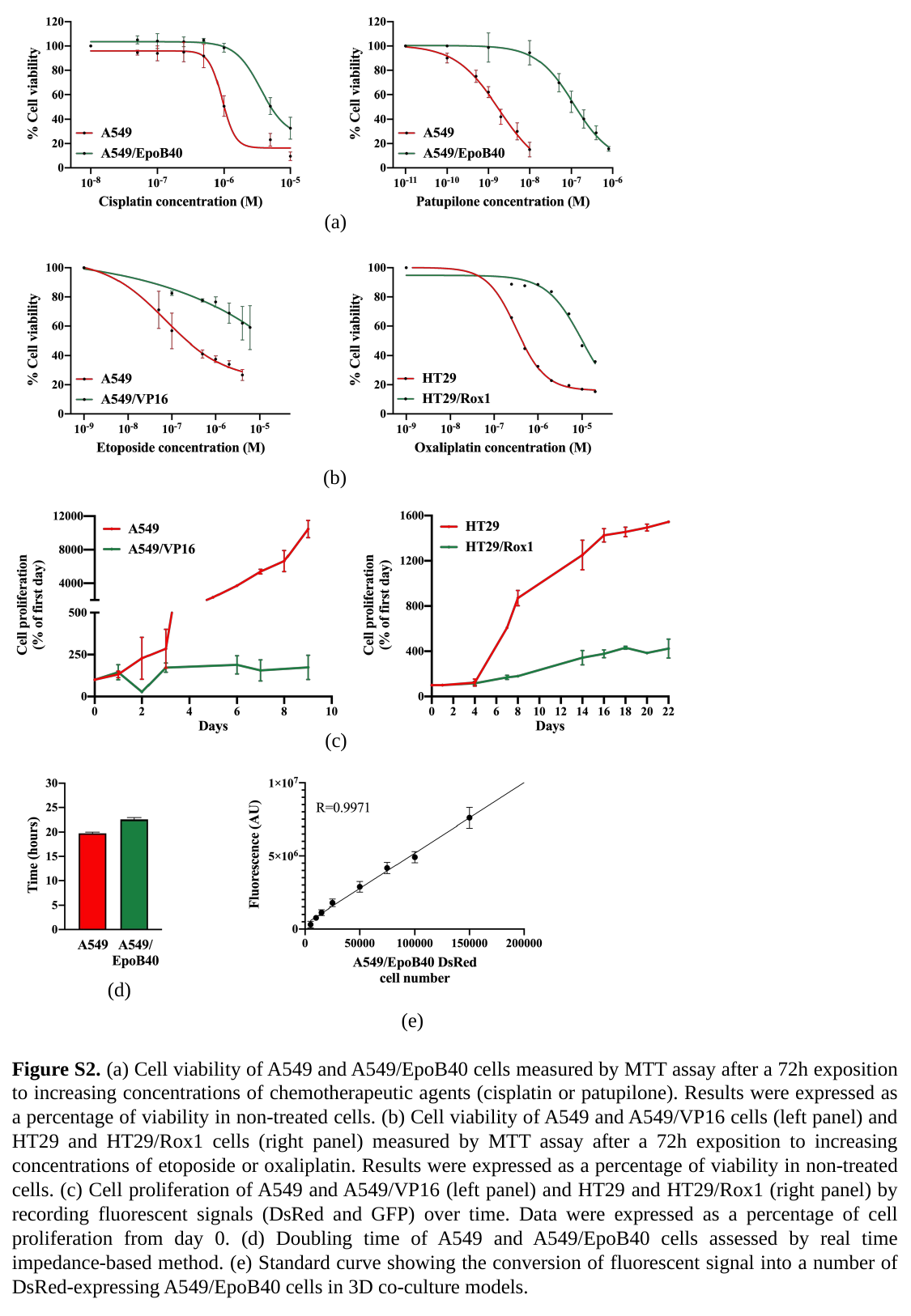

(a)
(b)
(c)
(d)
(e)
Figure S2. (a) Cell viability of A549 and A549/EpoB40 cells measured by MTT assay after a 72h exposition to increasing concentrations of chemotherapeutic agents (cisplatin or patupilone). Results were expressed as a percentage of viability in non-treated cells. (b) Cell viability of A549 and A549/VP16 cells (left panel) and HT29 and HT29/Rox1 cells (right panel) measured by MTT assay after a 72h exposition to increasing concentrations of etoposide or oxaliplatin. Results were expressed as a percentage of viability in non-treated cells. (c) Cell proliferation of A549 and A549/VP16 (left panel) and HT29 and HT29/Rox1 (right panel) by recording fluorescent signals (DsRed and GFP) over time. Data were expressed as a percentage of cell proliferation from day 0. (d) Doubling time of A549 and A549/EpoB40 cells assessed by real time impedance-based method. (e) Standard curve showing the conversion of fluorescent signal into a number of DsRed-expressing A549/EpoB40 cells in 3D co-culture models.

## Slide 3
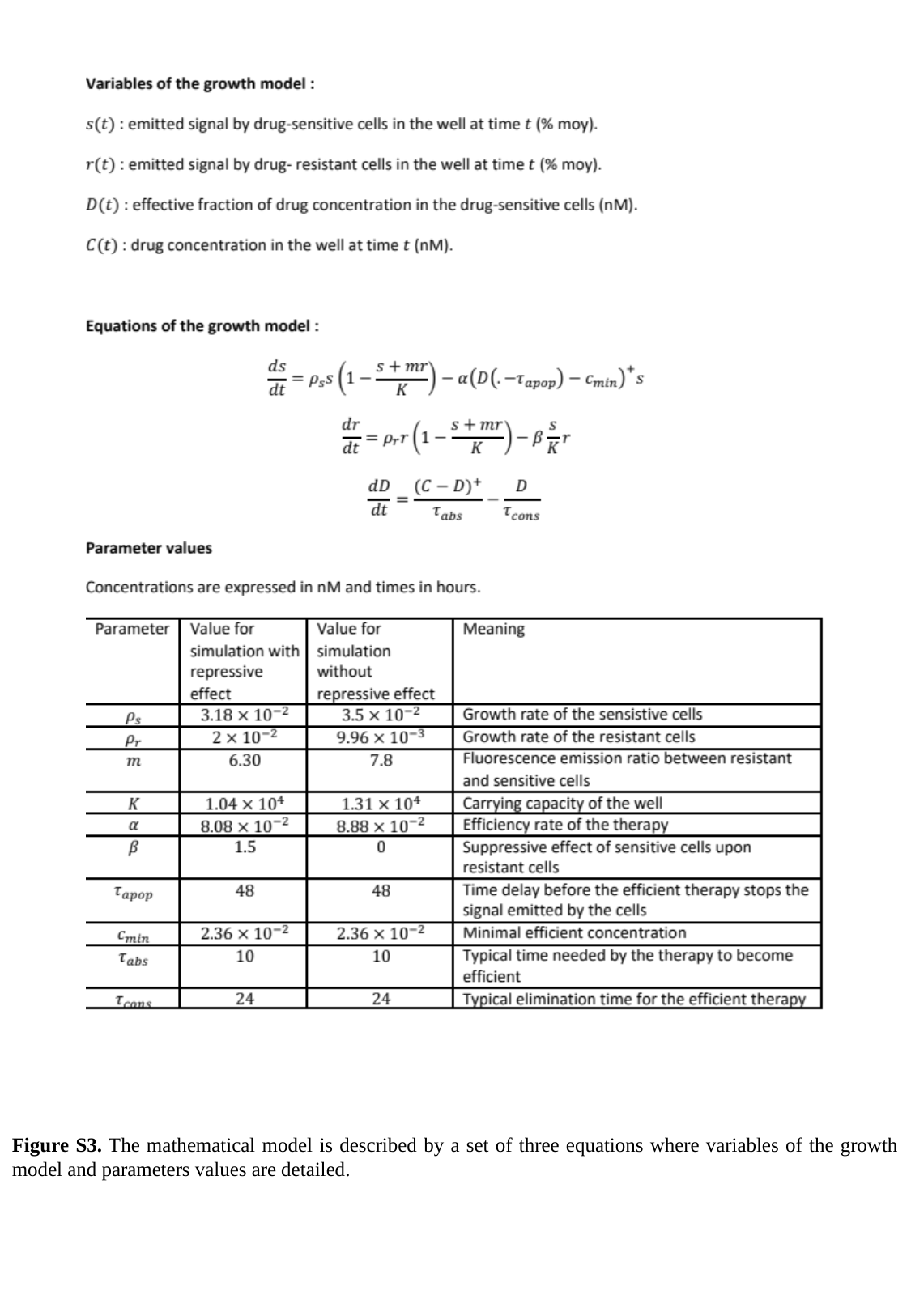

Figure S3. The mathematical model is described by a set of three equations where variables of the growth model and parameters values are detailed.

## Slide 4
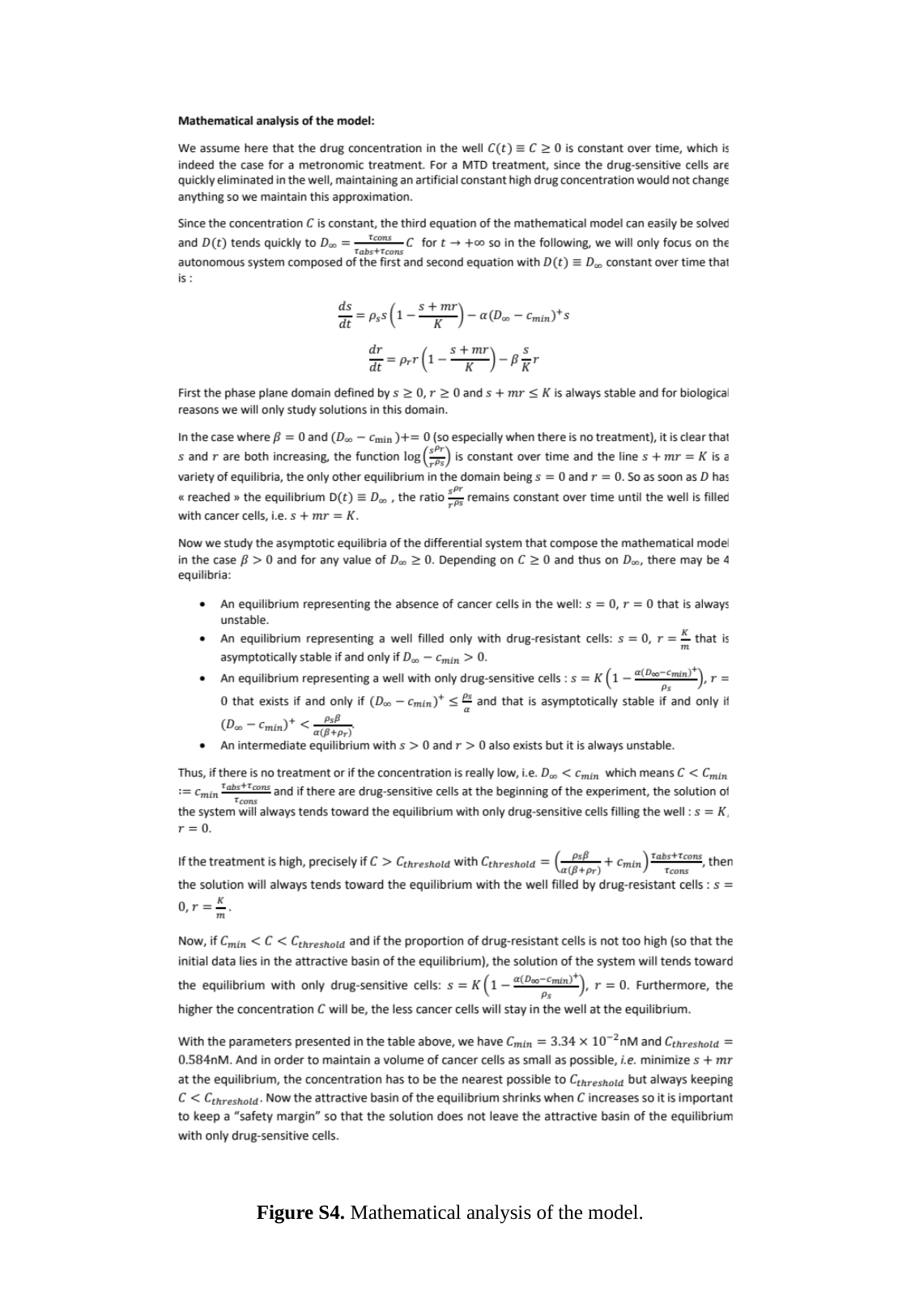

Figure S4. Mathematical analysis of the model.

## Slide 5
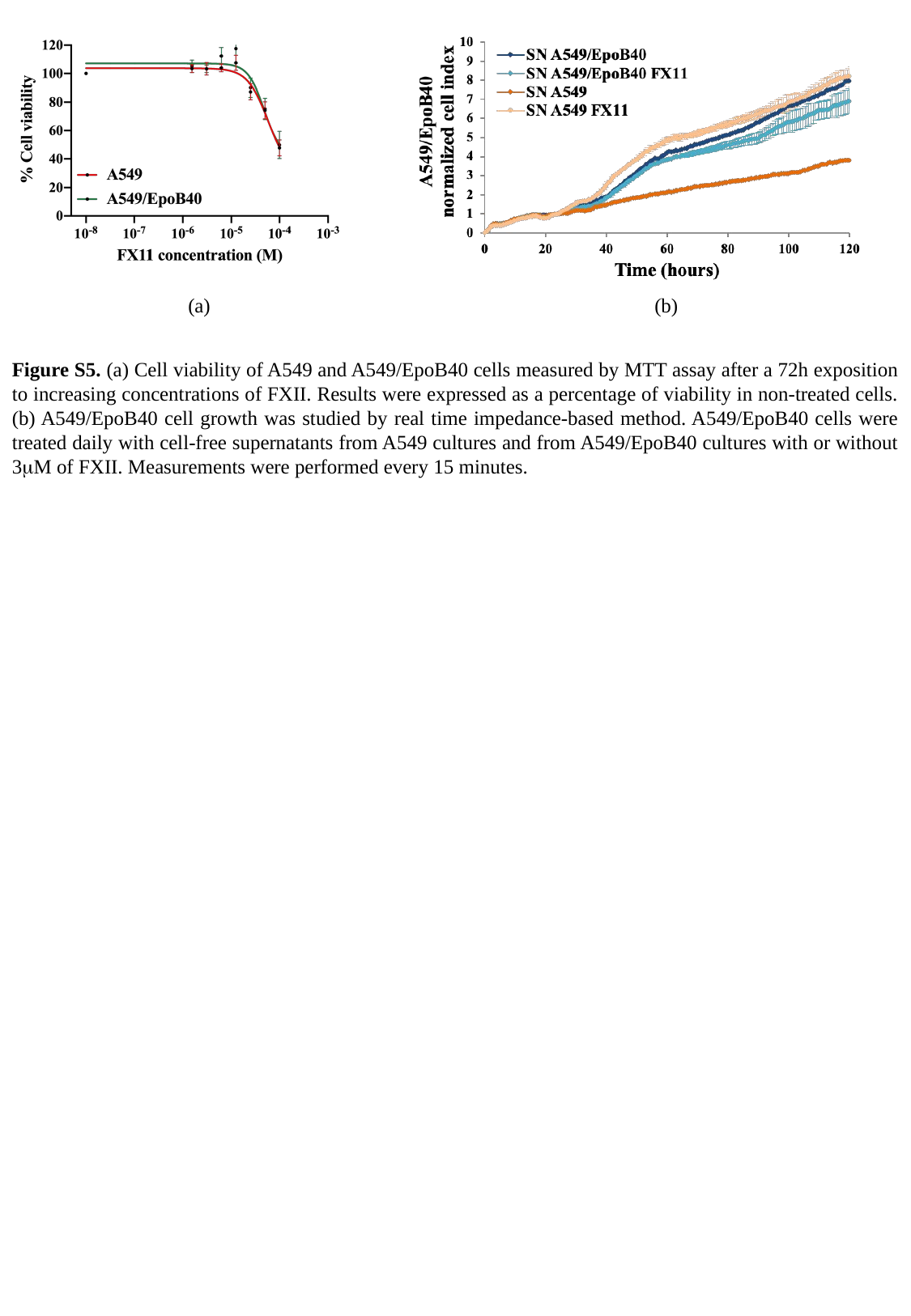

(a)
(b)
Figure S5. (a) Cell viability of A549 and A549/EpoB40 cells measured by MTT assay after a 72h exposition to increasing concentrations of FXII. Results were expressed as a percentage of viability in non-treated cells. (b) A549/EpoB40 cell growth was studied by real time impedance-based method. A549/EpoB40 cells were treated daily with cell-free supernatants from A549 cultures and from A549/EpoB40 cultures with or without 3μM of FXII. Measurements were performed every 15 minutes.
